# Potential role of TNFRSF12A in linking glioblastoma and alzheimer’s disease via shared tumour suppressor pathways

**DOI:** 10.1038/s41598-025-08000-7

**Published:** 2025-07-01

**Authors:** Ting Liu, Jingjing Pu, Sandra Theil, Yanxia Liu, Liping Jiang, Hongde Liu, Jarek Maciaczyk, Ingo G. H. Schmidt-Wolf, Jochen Walter, Amit Sharma

**Affiliations:** 1 Department of Geriatrics, Renji Hospital, Shanghai Jiao Tong University School of Medicine, Shanghai, 200127 China; 2 Department of Integrated Oncology, Center for Integrated Oncology (CIO) Bonn, University Hospital Bonn, 53127 Bonn, Germany; 3 Department of Hematology, Renji Hospital, Shanghai Jiao Tong University School of Medicine, Shanghai, 200127 China; 4 Molecular Cell Biology, Center of Neurology, University Hospital Bonn, 53127 Bonn, Germany; 5 Wuxi Maternity and Child Health Care Hospital, Wuxi School of Medicine, Jiangnan University, Wuxi, 214002 China; 6 State Key Laboratory of Bioelectronics, School of Biological Science & Medical Engineering, Southeast University, Nanjing, 210096 China; 7 Department of Stereotactic and Functional Neurosurgery, University Hospital Bonn, 53127 Bonn, Germany

**Keywords:** Tumor suppressor genes, Alzheimer’s disease, Glioblastoma, Machine learning, scRNA-seq, Cancer, Computational biology and bioinformatics, Genetics, Neuroscience, Neurology, Oncology

## Abstract

Tumor suppressor genes (TSGs) are critical regulators of cellular homeostasis and are extensively studied in cancer biology. However, their roles in neurodegenerative diseases, particularly Alzheimer’s disease (AD), remain poorly understood. Recent evidence of an inverse association between cancer and AD suggests the existence of shared molecular mechanisms. We conducted an integrative analysis to identify TSGs with potential involvement in both AD and glioblastoma (GBM), using Mendelian randomization, transcriptomic profiling (bulk and single-cell RNA-seq), cell–cell communication inference, and in vitro validation. Among 1,217 TSGs screened, *TNFRSF12A* was consistently dysregulated in both GBM and AD datasets. Further analysis revealed its association with immune-related pathways and transcriptional programs relevant to both diseases. Knockdown of *TNFRSF12A* in glioma cells altered the expression of genes associated with amyloid precursor protein (APP) processing and Wnt signaling pathways. This study identifies *TNFRSF12A* as a cross-disease candidate gene in GBM and AD, based on transcriptomic convergence and partial functional validation. Our findings suggest that TSGs may contribute to shared molecular programs in neurodegeneration and cancer, and warrant further mechanistic investigation.

**Supplementary Information:**

The online version contains supplementary material available at 10.1038/s41598-025-08000-7.

## Introduction

TSGs are essential regulators of cellular homeostasis and are widely studied in the context of cancer. However, their roles in neurodegenerative diseases (NDDs) remain poorly understood. Recent studies have highlighted a surprising inverse relationship between cancer and NDDs, including AD, whereby individuals with a history of cancer exhibit a reduced risk of developing AD, and vice versa^1–5^. These findings suggest the existence of shared molecular mechanisms that govern both diseases, despite their opposing cellular fates—uncontrolled proliferation in cancer versus progressive neuronal loss in AD^6–8^.

Increasing evidence points to common genes and signaling pathways linking these pathologies^6,9–13^. For example, TP53 (encoding p53) is not only a central regulator of the DNA damage response and apoptosis in cancer but has also been implicated in neuronal death and synaptic dysfunction in AD^14–16^. Other regulators, including PARK7, LRRK2, specific microRNAs, deubiquitinating enzymes, and adenosine receptors, have been shown to function at the intersection of tumor biology and neurodegeneration^17,18^. These observations underscore the need to re-evaluate the role of TSGs beyond oncology, particularly in AD, where their contribution remains largely uncharacterized.

Notably, evidence for a cancer–AD inverse relationship has grown rapidly in recent years, strengthening the rationale to investigate genes with dual roles in these diseases^16,19–21^. In this study, we focused on GBM and AD, two central nervous system (CNS) disorders at opposite ends of the cell fate spectrum, to explore potential links between tumor suppression and neurodegeneration.

**Fig. 1:**
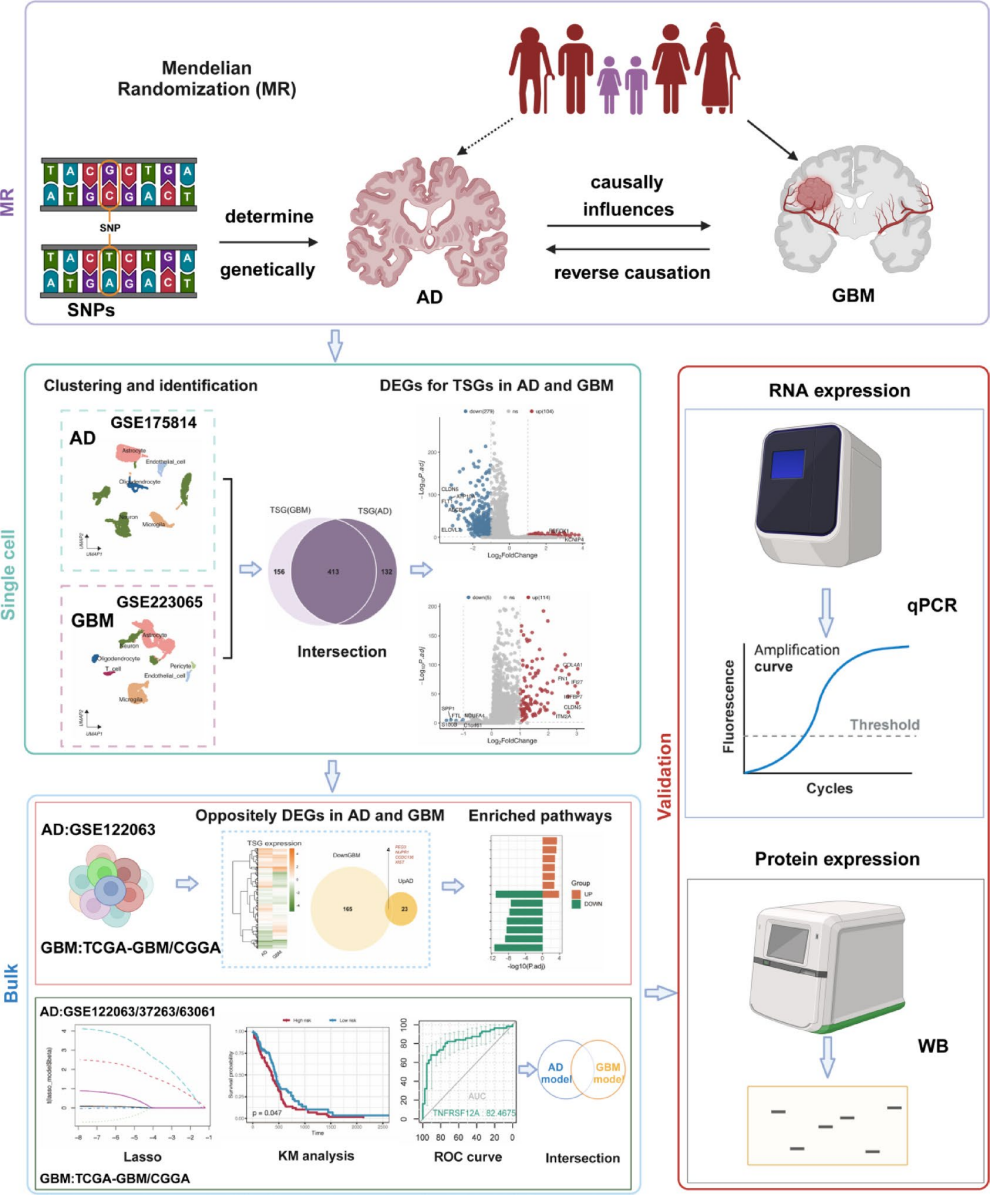
Overview of the workflow for the causal relationship between AD and GBM, dataset analysis, and experimental validation.

To address this, we conducted a multi-layered analysis integrating Mendelian randomization (MR) to assess potential causal relationships^22–25^, bulk and single-cell RNA sequencing to examine expression dynamics, cell–cell communication analysis to reveal signaling roles, and in vitro knockdown assays to explore functional impact. The flowchart of our study is shown in Fig. 1**.**

We systematically analyzed the expression profiles of 1217 known TSGs across transcriptomic datasets from AD and GBM. This approach enabled us to identify both overlapping and oppositely regulated candidates, among which *TNFRSF12A* consistently showed differential expression in both conditions.

Our integrative framework prioritized *TNFRSF12A* as a potential cross-disease candidate, suggesting its possible involvement in shared biological processes between tumorigenesis and neurodegeneration. These findings contribute to a broader understanding of the transcriptional landscape of TSGs in central nervous system disorders and provide a basis for future functional and translational investigations.

## Results

### Causal effects between AD and GBM

To explore potential causal links between AD and GBM, we conducted bidirectional two-sample MR using SNPs as instrumental variables. Summary statistics were obtained from GWAS datasets covering European populations: 17,008 AD cases and 37,154 controls, 91 GBM cases and 174,006 controls, and 407,089 individuals with a parental history of AD (Table 1).

**Table 1:** An overview of the data for MR analysis.

	Dataset	#SNPs in GWAS	Population	Cases	Controls	Year	PubMed ID
GBM	finn-b-C3_GBM_EXALLC	16,380,303	European	91	174,006	2021	
AD	ebi-a-GCST002245	7,022,150	European	17,008	37,154	2013	24,162,737
Parental history of AD	ebi-a-GCST90013921	11,039,079	European	407,089		2021	34,017,140

**Fig. 2:**
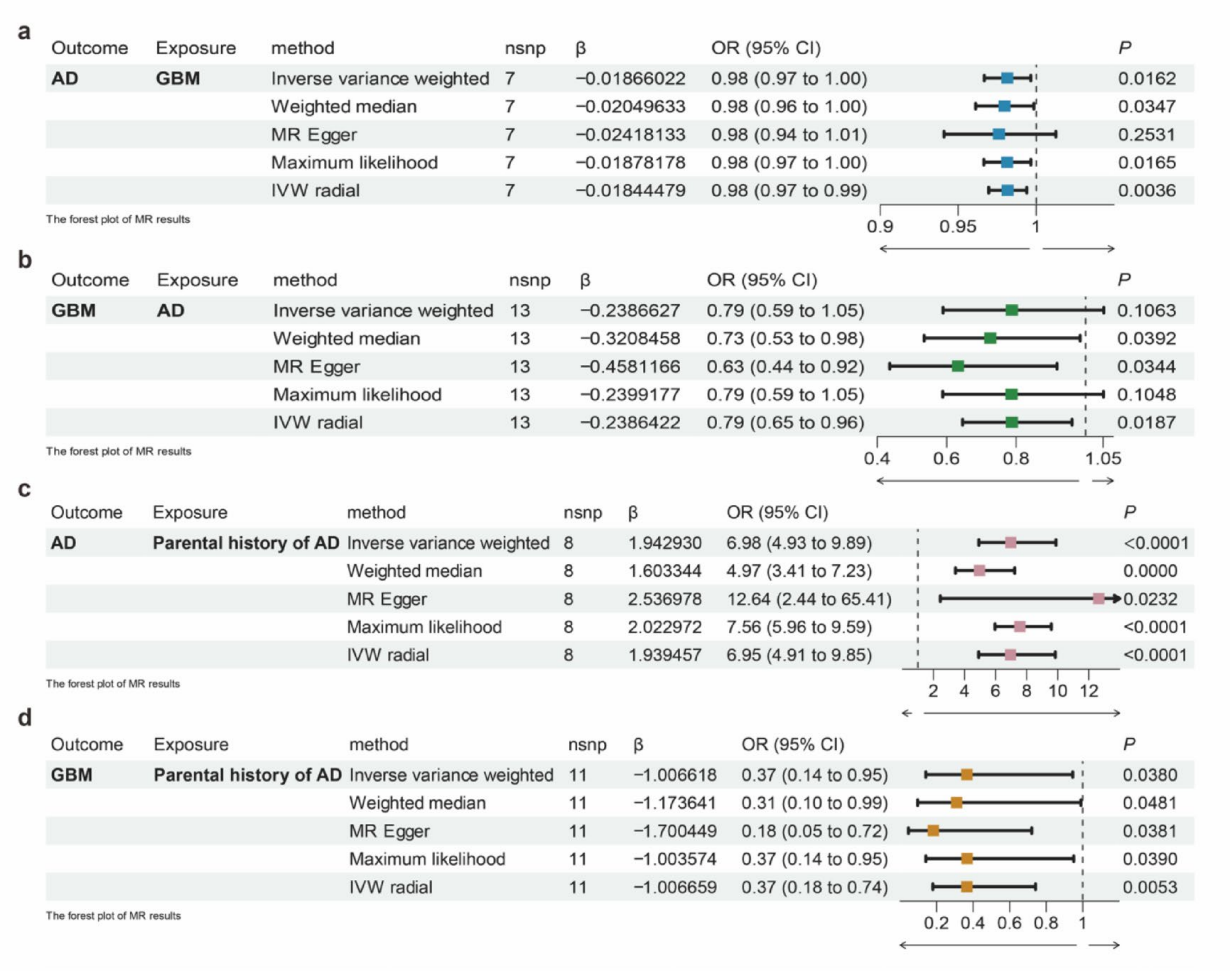
Mendelian randomization analysis of the causal relationships among AD, GBM and Parental history of AD. (**a**) Forest plot showing causal estimate of GBM on AD. (**b**) Forest plot showing causal estimate of AD on GBM. (**c**) Forest plot showing causal estimate of Parental history of AD on AD. (**d**) Forest plot showing causal estimate of Parental history of AD on GBM. The odds ratio (OR) was estimated using the Inverse variance weighting, Weighted median, MR-Egger regression, Maximum likelihood and IVW radial. The horizontal bars represent 95% confidence intervals (CI).

The MR estimates from different methods are shown in Fig. 2. In the forward direction (GBM as exposure, AD as outcome), multiple MR methods consistently suggested a modest but statistically significant inverse causal relationship. The inverse variance weighting (IVW) method yielded an odds ratio (OR) of 0.98 (95% CI: 0.97–1.00, *P* = 0.0162), supported by weighted median (*P* = 0.0347) and maximum likelihood estimates (*P* = 0.0165). The radial IVW method further confirmed the association with improved statistical power (*P* = 0.0036) (Fig. 2a). Although MR-Egger showed a similar direction of effect, its result did not reach statistical significance (*P* = 0.2531).

Reverse MR analysis (AD as exposure, GBM as outcome) showed a similar trend. The weighted median and MR-Egger methods reported significant associations with ORs of 0.73 (*P* = 0.0392) and 0.63 (*P* = 0.0344), respectively, while IVW (*P* = 0.1063) and maximum likelihood (*P* = 0.1096) did not reach statistical significance. The IVW radial method also indicated a significant inverse association (OR = 0.79, 95% CI: 0.65–0.96, *P* = 0.0187) (Fig. 2b).

To further validate this relationship, we used parental history of AD as an alternative proxy for genetic susceptibility to AD. This trait showed a strong positive causal association with AD risk (IVW OR = 6.98, 95% CI: 4.93–9.89, *P* < 0.0001) and a significant inverse association with GBM risk (IVW OR = 0.37, 95% CI: 0.14–0.95, *P* = 0.0380) (Fig. 2c–d).

Heterogeneity and horizontal pleiotropy were assessed to ensure the validity of instrumental variables. Cochran’s Q test and MR-Egger intercepts indicated low heterogeneity and no directional pleiotropy in most models, particularly in GBM–AD and GBM–parental AD analyses. However, modest heterogeneity was observed in the parental AD –AD comparison (Table 2), suggesting potential complexity in underlying pathways.

**Table 2:** Observations from Heterogeneity and Pleiotropy Tests in Mendelian Randomization analysis.

Outcome	Exposure	Heterogeneity test	Q	Q_df	Q_*P*val	Egger_intercept	*P*val
AD	GBM	MR Egger	3.81943321	5	0.57569505	0.006197692	0.7590844
		Inverse variance weighted	3.92438015	6	0.68690922		
GBM	AD	MR Egger	2.26205198	11	0.99734307	0.1269191	0.09245345
		Inverse variance weighted	5.65786487	12	0.93233771		
AD	Parental history of AD	MR Egger	63.4257537	8	9.87E-11	-0.03659904	0.1705198
		Inverse variance weighted	81.4042207	9	8.50E-14		
GBM	Parental history of AD	MR Egger	3.65474526	9	0.93263348	0.1032982	0.2029453
		Inverse variance weighted	5.54026893	10	0.85229728		

Together, these results indicate a statistically supported and biologically plausible inverse causal relationship between AD and GBM. Genetic predisposition to one condition appears to confer reduced risk for the other, implicating shared yet potentially antagonistic mechanisms at the genomic level.

### Fundamental and systems-level variations in cell communication and signaling between AD and GBM

**Fig. 3:**
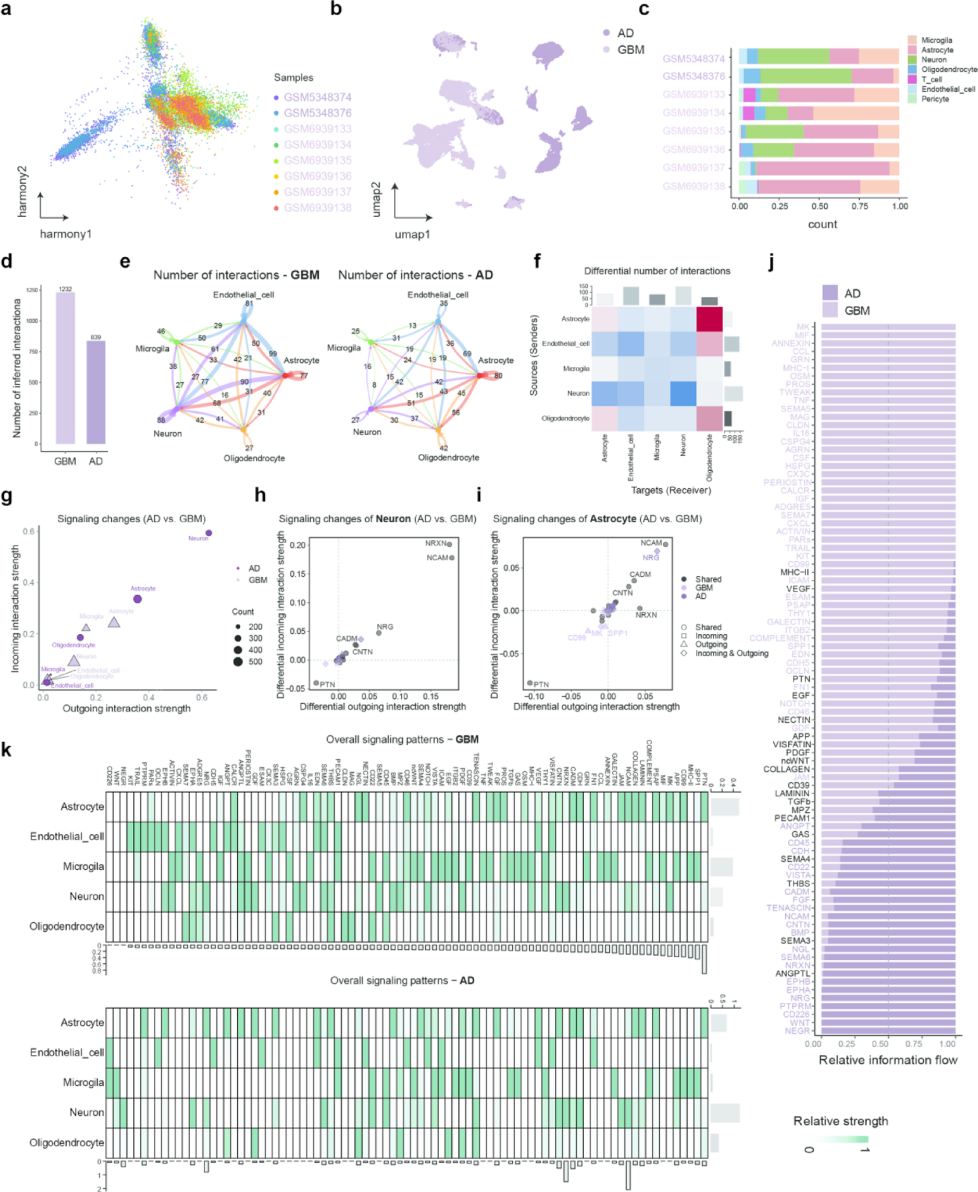
scRNA-seq analysis reveals altered cell–cell communication in AD versus GBM. (**a**, **b**) The integration effect of AD and GBM. c The distribution of cell types derived from AD and GBM patients. (**d**) Total number of possible interactions. (**e**) Number of possible interactions between the five major cell types of AD and GBM. (**f**) Differential number of possible interactions between any two cell populations. Red (high in AD) and blue (high in GBM) in the color bar indicate the number of predicted interactions in AD and GBM, respectively. (**g**) Differential interaction analysis identifying prominently altered signaling sources and targets. (**h**, **i**) Altered Neurons and Astrocytes signaling pathways in AD versus GBM. The x and y axes represent differential outgoing and incoming interaction strengths, respectively. Positive and negative values indicate increased and decreased signaling, respectively, in AD compared with GBM. Dot shapes indicate whether a signaling pathway is specific to either AD or GBM in its outgoing, incoming, or both outgoing and incoming signaling. (**j**) Significant signaling pathways were ranked based on their differences of overall information flow, calculated by summarizing all communication probabilities in a given inferred network. Those colored dark purple and light purple are more enriched in AD and GBM, respectively. (**k**) The strength of interaction of significant signalling pathways is rated based on their differences in overall information flow across cell types in AD and GBM.

The Harmony algorithm was employed to correct batch effects in two scRNA-seq datasets (GSE175814 and GSE223065) (Fig. 3a,b). This scRNA-seq profiling was undertaken to delineate the cellular landscapes in AD and GBM. Heatmaps (Supplementary Fig. 1a,b) depicted the expression profiles of marker genes across these cell types, corroborating their identities and functional roles. The Uniform Manifold Approximation and Projection (UMAP) analysis revealed distinct cellular landscapes in AD and GBM tissues. In AD, five major cell types were identified—astrocytes, neurons, microglia, oligodendrocytes, and endothelial cells (Supplementary Fig. 1c). In contrast, GBM samples exhibited a more complex cellular composition, with seven cell types detected, including pericytes and T cells in addition to the five found in AD (Supplementary Fig. 1d). Astrocytes, neurons, and microglia were the predominant populations in both conditions (Supplementary Fig. 1c,d and Fig. 3c), highlighting both shared and disease-specific cellular architectures. Notably, each cell type displayed distinct levels of TSG expression, regardless of disease context (Supplementary Fig. 1e,f), suggesting cell type–specific regulation of these genes in the central nervous system.

To understand how intercellular signaling varies between AD and GBM, we analyzed single-cell RNA-seq datasets from AD (GSE175814) and GBM (GSE223065) using the CellChat framework. This approach allowed inference of ligand–receptor-mediated interactions across major brain cell types, including astrocytes, neurons, microglia, oligodendrocytes, and endothelial cells. GBM had a greater number of potential interactions compared to AD (Fig. 3d). In GBM, there was an observed increase in signalling among astrocytes, neurons, and endothelial cells, but signalling between astrocytes and oligodendrocyte reduced (Fig. 3e, f). Subsequently, we conducted a study on network centrality to examine the changes in signalling patterns between AD and GBM. This technique calculates the strength of interactions, both outgoing and incoming, for each cell subpopulation in order to determine their probability as sources and targets of signalling, respectively. In general, the signalling activity of neurons and astrocytes increases in AD, either in terms of outgoing or incoming signals (Fig. 3g). The analysis of the communication between neurons and astrocytes in AD showed a significant rise in both incoming and outgoing signals for neural cell adhesion molecule (NCAM), neuregulin (NRG), cell adhesion molecule (CADM), and contactin (CNTN) (Fig. 3h, i); Conversely, there was a decrease in both incoming and outgoing signals for pleiotrophin (PTN) when compared to GBM (Fig. 3h, i). We subsequently examined the information flow, which is determined by the total communication probability between all pairs of cell groups in the predicted network, for distinct signalling pathways in AD vs. GBM. Neuronal growth regulator (NEGR), wingless-related integration site (WNT), and DNAM-1 (CD226) exhibited a significant increase only in AD (Fig. 3j, k). Midkine (MK), macrophage migration inhibitory factor (MIF), chemokine (C–C motif) ligand (CCL), granulin precursor (GRN), major histocompatibility complex class I (MHC-I), and oncostatin M (OSM) exhibited a significant rise in their information flow only in GBM (Fig. 3j, k). Surprisingly, this finding highlights unexpected and complex dynamics in disease-specific signaling pathways within microglial cells, which are central to immune responses in the brain. The APP signalling pathway exhibited a greater degree of interaction in the microglia population of GBM (Fig. 3j, k). This could imply a previously unrecognized role of APP in tumor biology or immune modulation in GBM^26–29^. Conversely, the Wnt pathway, which is frequently activated in GBM, was only activated in AD with higher interaction strength in microgila population (Fig. 3j, k). This unusual activation in AD suggests that microglial signaling in neurodegeneration may be more closely linked to pathways involved in cancer biology, indicating overlapping molecular mechanisms between neurodegeneration and tumorigenesis.

When these results are combined with MR findings, a mutual relationship between the genetic factors of GBM and AD becomes apparent. These findings provide evidence for the concept of both shared and conflicting genetic factors in the development of GBM and AD.

### scRNA-seq analysis of shared and differential TSGs expression between AD and GBM

After confirming a genetic inverse association between AD and GBM, we next investigated the transcriptional activity of TSGs at single-cell resolution. TSGs are critical in regulating cell growth, genomic stability, and intercellular communication^30^. In GBM, they are often activated early as protective responses but become silenced or bypassed during tumor progression^31,32^. In contrast, in AD, TSGs may play a role in regulating neuronal survival and preventing neurodegeneration, potentially influencing pathways involved in cell signaling and communication^33^. By comparing shared and disease-specific expression profiles of TSGs, we aimed to uncover potential mechanisms underlying these opposing pathological processes.

**Fig. 4:**
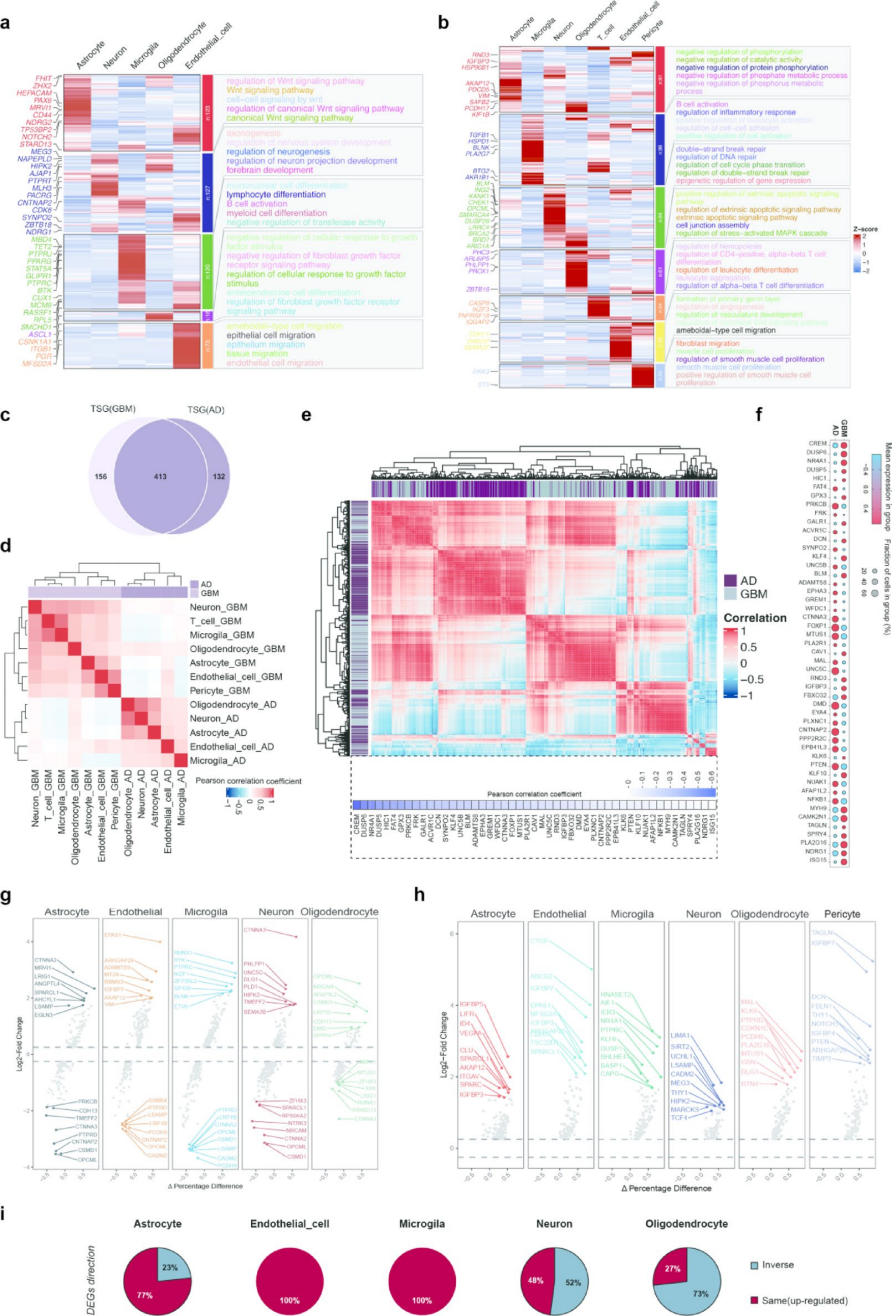
Shared and distinct TSG expression profiles in AD and GBM. (**a**) Expression of TSGs in each cell type with corresponding enriched pathways (GO_BP) in AD. (**b**) Expression of TSGs in each cell type with corresponding enriched pathways (GO_BP) in GBM. (**c**) TSGs shared between AD and GBM scRNA-seq datasets. (**d**) The Pearson correlation coefficient was calculated to compare the expression of 413 TSGs between AD and GBM across different cell types. (**e**) The Pearson correlation coefficient was calculated to compare the expression of 413 TSGs between AD and GBM. The TSGs that exhibited a negative correlation between AD and GBM are displayed in the heatmap below. (**f**) The mean expression of negatively correlated TSGs in both AD and GBM scRNA-seq datasets is shown. (**g**, **h**) The volcano plot displays shared TSGs that are either up- or down-regulated in AD (**g**) or GBM (**h**) across different cell types. i The comparison of DEGs direction in GBM and AD across cell types.

Intersection analyses (Fig. S1e, f) highlighted cell types predominantly expressing TSGs, which comprised approximately 10% of the total in both AD and GBM, suggesting critical cellular targets. The TSGs within each cell type were associated with distinct enriched biological processes in AD (Fig. 4a) and GBM (Fig. 4b), providing insights into their roles in disease mechanisms. In astrocytes, TSGs were enriched in the Wnt signaling pathway in AD, whereas they were involved in the negative regulation of phosphorylation predominantly in GBM (Fig. 4a, b). In neurons, TSGs were linked to nervous system development in AD but were associated with brake repair and DNA repair mechanisms in GBM (Fig. 4a, b). In endothelial cells, TSGs were primarily mapped to cell migration in AD, while in GBM, they were associated with angiogenesis and vasculature development (Fig. 4a, b). A total of 413 TSGs were recognized as shared genes in the AD and GBM datasets (Fig. 4c), indicating the presence of common molecular foundations. The Pearson correlation analysis investigated the expression of shared TSGs between AD and GBM. The study revealed negative correlations between the majority of cell types in AD and GBM, with approximately half of the TSGs also exhibiting negative correlations between the two diseases (Fig. 4d, e). The negative correlations between cell types in AD and GBM are consistent with the inverse relationship revealed by MR and distinct signaling dynamics from cell communication analyses, reflecting opposing mechanisms of neurodegeneration and tumorigenesis. A total of 49 shared TSGs were discovered to exhibit a negative correlation between AD and GBM (Fig. 4e). The mean expression level of TSGs that were negatively correlated was likewise found to be in the opposite direction (Fig. 4f). The volcano plots (Fig. 4g, h) exhibited prominently up- and down-regulated shared TSGs in both AD and GBM. In AD, the distribution of shared TSGs was strongly shifted either upwards or downwards (Fig. 4g). While in GBM, the differentially expressed TSGs showed restricted trend of upregulated across cell types (Fig. 4h). Next, we investigated the possibility of an inverse trend of DEGs, i.e. TSGs that are highly expressed in GBM and the same cluster of TSGs being low-expressed in AD. The analysis revealed that astrocytes (23%), neurons (52%) and oligodendrocytes (73%) harbor TSGs with an inverse pattern of being high in GBM and low in AD (Fig. 4i).

TSG score in AD (Supplementary Fig. 2a) and GBM (Supplementary Fig. 2b) was calculated for each cell using the AUCell R package. Cells were categorized into high and low TSG score groups based on the median score. Notably, there was essentially no cell type with high TSG score in AD (Supplementary Fig. 2a). The volcano plots illustrated a distinct differentiation in gene expression profiles between the low and high TSG score groups in AD (Supplementary Fig. 2c) and GBM (Supplementary Fig. 2d). Gene Set Enrichment Analysis (GSEA) revealed significant differences in the enrichment of various biological processes between the low and high TSG score groups in AD (Supplementary Fig. 2e). The upregulated pathways in the low TSG score group indicated substantial alterations in metabolic and immune responses, cell proliferation, and transport mechanisms. The top three upregulated KEGG pathways in the low TSG score group in AD were neuroactive ligand-receptor interaction, calcium signaling pathway, and long-term depression (Supplementary Fig. 2f). Conversely, the top three downregulated KEGG pathways were ribosome, focal adhesion, and cytokine receptor interaction in AD (Supplementary Fig. 2g). In GBM, the high TSG score group (Supplementary Fig. 2h) showed downregulation in processes related to cell activation, phosphorylation, and biosynthesis. However, pathways related to circulatory system development, morphogenesis, and transport were upregulated. The top three upregulated KEGG pathways in the high TSG score group in GBM were focal adhesion, ECM-receptor interaction, and pathways in cancer (Supplementary Fig. 2i). The top three downregulated KEGG pathways were oxidative phosphorylation, AD, and ribosome in GBM (Supplementary Fig. 2j).

Together, these results demonstrate a fundamental divergence in TSG activity between AD and GBM. In AD, low TSG activity is linked to alterations in metabolic and neuronal signaling pathways, potentially reflecting degenerative changes. In contrast, elevated TSG expression in GBM appears paradoxically associated with proliferative, angiogenic, and cancer-supportive processes. These findings further support the notion of inverse regulation of TSGs in neurodegeneration and tumorigenesis.

### Inverse regulation of TSGs in bulk cohort analysis between AD and GBM

**Fig. 5:**
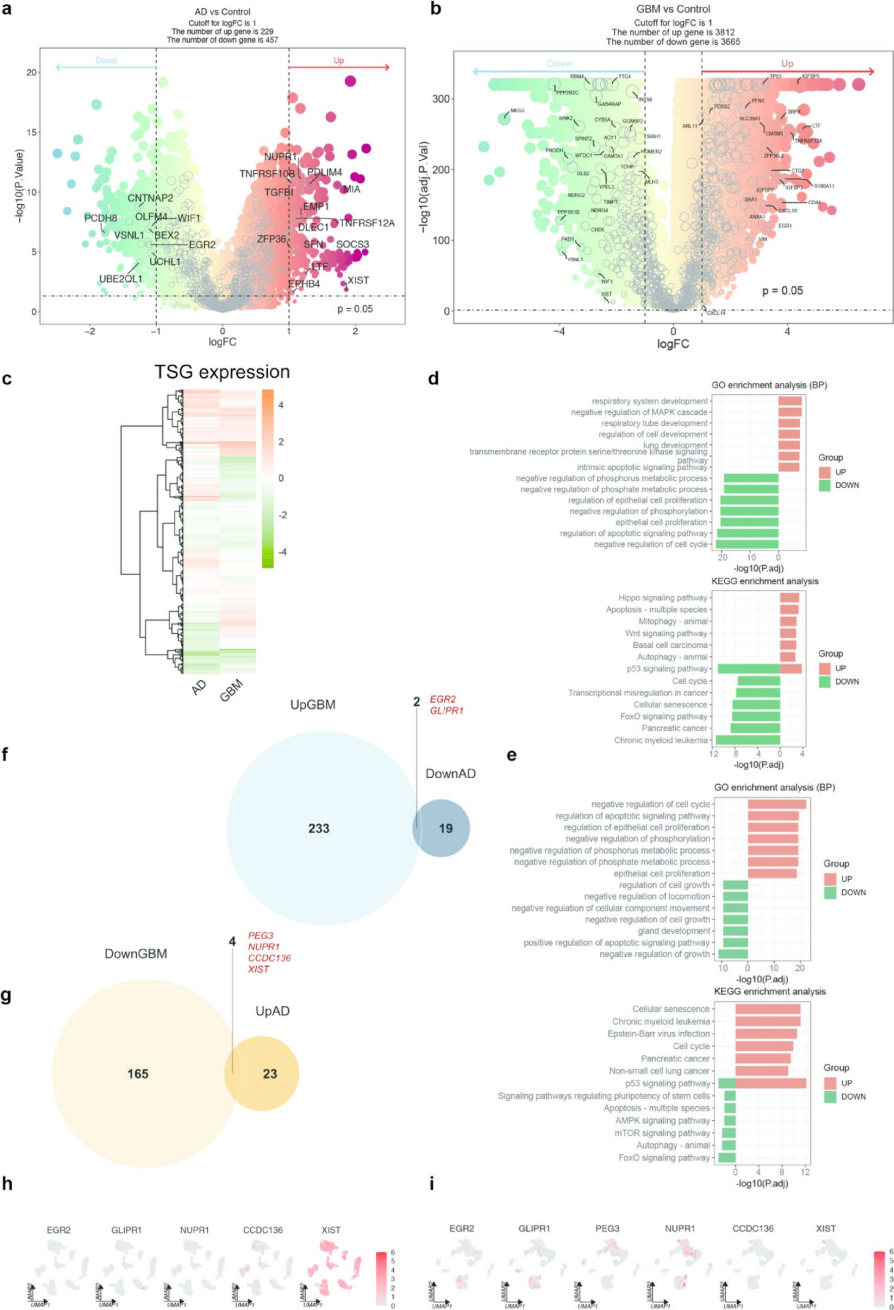
Inverse regulation of TSGs in bulk cohort analysis between AD and GBM. (**a**) DEGs between AD and healthy control (HC) in the bulk cohort (GSE122063), with the distribution of TSGs denoted by gray circles. (**b**) DEGs between GBM and HC in the TCGA bulk cohort (TCGA-GBM), with the distribution of TSGs denoted by gray circles. (**c**) The expression pattern of TSGs in both AD and GBM (bulk cohorts). (**d**) Bar plots depicting the enrichment analysis of TSGs in AD through GO and KEGG pathways according to the fold change, with measured significance by -log10(adj.*P*.Val). (**e**) Bar plots depicting the enrichment analysis of TSGs in GBM through GO and KEGG pathways according to the fold change. (**f**, **g**) The Venn diagram illustrates DEGs that exhibit inverse expression trends in AD and GBM comparing to their respective HC. (**h**, **i**) Confirming the scRNA-seq expression profiles of selected genes (such as *EGR2*, *GLIPR1*, *PEG3*) that display reverse expression trends in AD and GBM.

Following the identification of overlapping TSG expression patterns in the scRNA-seq data, we extended our investigation to Bulk RNA-seq analysis. This analysis was performed to compare TSG expression profiles in AD and GBM relative to healthy controls (HC). Only few TSGs were differentially expressed in the AD cohort (GSE122063) (Fig. 5a), while a larger number were differentially expressed in the TCGA-GBM cohort (Fig. 5b). Approximately half of the TSGs exhibited inverse trend (opposite expression patterns) between AD and GBM (Fig. 5c). GO and KEGG pathway analyses identified significant enrichment in both up- and downregulated TSGs, indicating critical biological processes and pathways involved in AD and GBM, and highlighting the distinct roles TSGs play in each condition (Fig. 5d, e). Specifically, development-related biological functions, apoptosis, mitophagy, and autophagy were upregulated in AD, while cell proliferation, cell senescence, and cell cycle processes were downregulated (Fig. 5d). These changes were exactly the opposite in GBM, where the same pathways exhibited inverse regulation (Fig. 5e). Specifically, *EGR2* and *GLIPR1* were upregulated in GBM but downregulated in AD compared to HC (Fig. 5f). Conversely, *PEG3*, *NUPR1*, *CCDC136*, and *XIST* were downregulated in GBM but upregulated in AD (Fig. 5g). The scRNA-seq dataset further illustrated these expression profiles. Notably, *XIST* showed markedly different expression levels between AD and GBM, with high expression across all cell types in AD (Fig. 5h) and low expression in GBM (Fig. 5i). The findings of Bulk RNA-seq indicate that TSGs demonstrate unique and frequently opposing expression patterns in AD and GBM. The contrasting expression patterns emphasize distinct biological processes, with AD demonstrating elevated development-related functions and autophagy, while GBM displays enhanced cell proliferation and cell cycle activity. Further functional studies will be essential to determine whether these inverse expression patterns contribute directly to disease processes or reflect broader differences in the cellular environment.

### Prognosis-related TSGs signature in AD

**Fig. 6:**
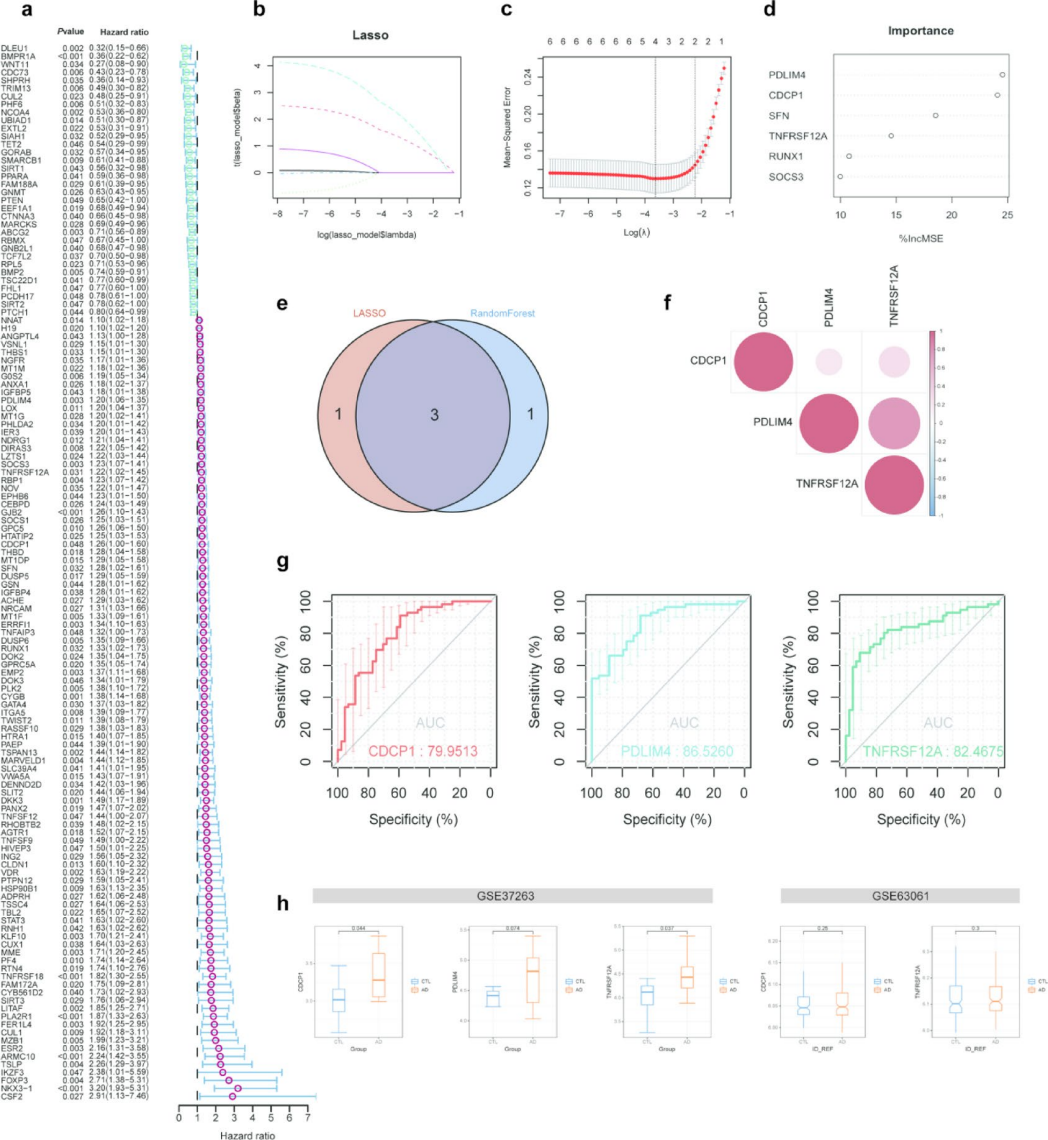
Prognosis-related TSGs signature in AD. (**a**) Identification of prognosis-related TSGs in GBM patients in the TCGA cohort. (**b**) Least absolute shrinkage and selection operator (LASSO) regression screening for AD-related TSG hallmark genes. (**c**) Construction of linear models (LASSO) and visualization by coefficients. (**d**) Importance ranking of all selected genes in random forest. (**e**) Venn diagram displays the shared TSGs signature obtained by two machine learning algorithms. (**f**) Spearman correlation analysis of the three TSGs. (**g**) ROC curves for calculating the signature genes’ diagnostic performance. (**h**) Characterization of TSGs signature expression in the external validation cohort (GSE37263/GSE63061).

Univariate cox regression analysis identified 138 TSGs with prognostic potential for GBM in the TCGA cohort (Fig. 6a). Among these, six TSGs were found to be upregulated and differentially expressed in AD bulk RNA-seq cohort. To identify key genes within these six TSGs in AD, two machine learning algorithms—LASSO and Random Forest—were employed (Fig. 6b–d). Three key genes, *CDCP1*, *PDLIM4*, and *TNFRSF12A*, were identified as significant by both methods (Fig. 6e). Notably, *PDLIM4* and *TNFRSF12A* exhibited a stronger correlation (Fig. 6f). The ROC analysis showed that *TNFRSF12A* had an AUC of 0.824, indicating high diagnostic accuracy in distinguishing AD patients from controls (Fig. 6g). This was comparable to *PDLIM4* (AUC = 0.865) and superior to *CDCP1* (AUC = 0.799). In the external validation cohort (GSE37263), the expression levels of these key genes were significantly higher in AD (Fig. 6h). Another external validation cohort (GSE63061) also showed an increasing trend in the expression levels of these key genes in AD (Fig. 6h). Therefore, the genes *CDCP1*, *PDLIM4*, and *TNFRSF12A*, due to their significant upregulation and high diagnostic performance, have the potential to aid in the early detection and prognosis of AD.

### Construction and validation of predictive models for TSGs in GBM

**Fig. 7:**
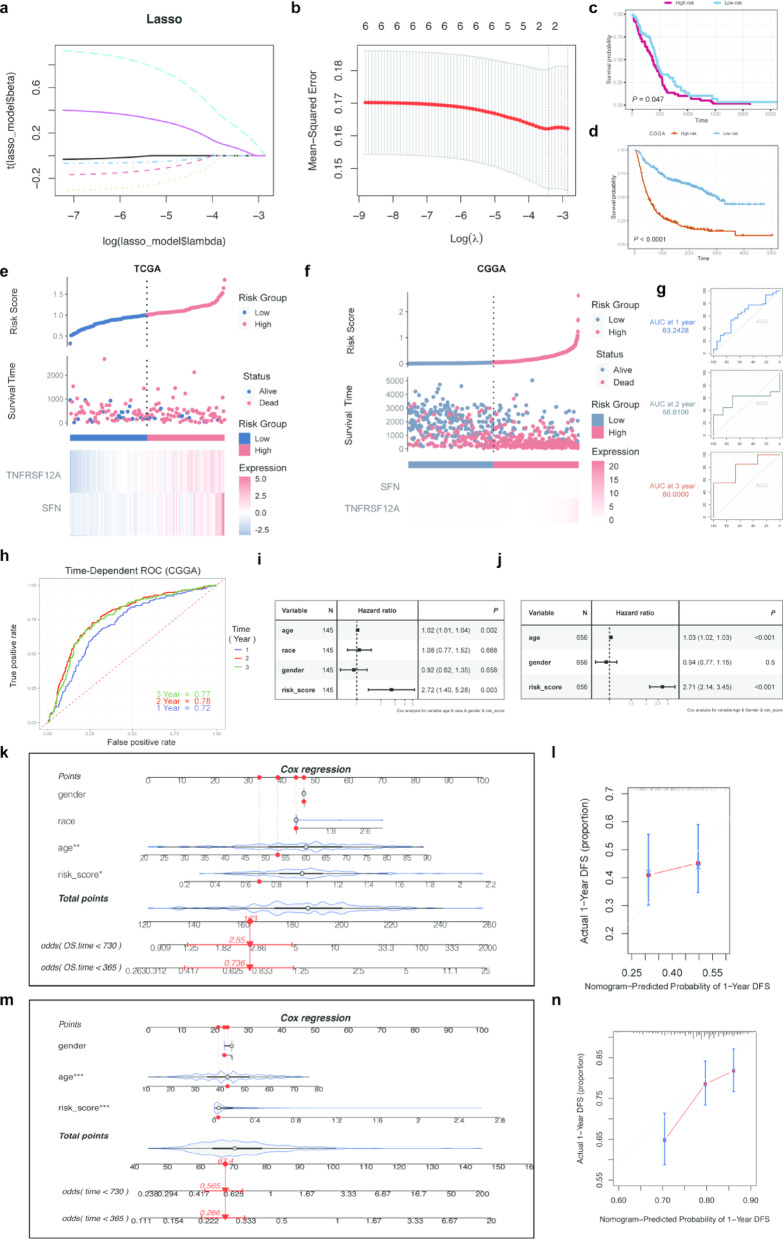
Constructing prognostic models for patients with GBM. (**a**, **b**) LASSO regressions obtained 2 prognosis-related TSGs. (**c**, **d**) Overall survival analysis based on the TSGs risk model in TCGA-GBM and CGGA cohorts. Patients with high risk had significantly worse prognoses than patients with low risk. (**e**, **f**) The risk plots of survival status of each sample in the TCGA and CGGA cohorts. Heat map showing the expression of each gene. (**g**, **h**) AUC values for TCGA and CGGA cohorts risk groupings at 1, 2, and 3 years. (**i**, **j**) Multivariate Cox analysis to assess the prognostic signature and clinical attributes in TCGA and CGGA cohorts such as age, race, and gender. (**k**) Nomogram of risk groupings and clinical characteristics for predicting survival at 1 and 2 years in the TCGA cohort. (**l**) Calibration curves for testing the agreement between actual and predicted outcomes at 1 year in the TCGA cohort. (**m**) Nomogram of risk groupings and clinical characteristics for predicting survival at 1 and 2 years in the CGGA cohort. (**n**) Calibration curves for testing the agreement between actual and predicted outcomes at 1 year in the CGGA cohort.

A risk-scoring model was developed using six TSGs identified in the previous analysis to pinpoint potential prognostic biomarkers for GBM. LASSO regression analysis revealed *TNFRSF12A* and *SFN* as independent prognostic factors (Fig. 7a, b). Risk scores were computed according to the methodology described. Based on the median risk score, patients in the TCGA and CGGA cohorts were categorized into high-risk and low-risk groups. Survival analysis showed that high-risk patients had significantly lower overall survival (OS) compared to low-risk patients in both the TCGA (Fig. 7c) and CGGA cohorts (Fig. 7d). Risk maps illustrated survival outcomes for the TCGA (Fig. 7e) and CGGA cohorts (Fig. 7f), while heat maps depicted expression variations of *TNFRSF12A* and *SFN* across different risk groups. The risk score predicted OS in the TCGA cohort for up to 3 years, with AUC values of 0.632, 0.568, and 0.8 for 1-, 2-, and 3-year OS, respectively (Fig. 7g). In contrast, the CGGA cohort, which had a more favorable prognosis for GBM patients, showed AUC values of 0.77, 0.78, and 0.72 for 1-, 2-, and 3-year OS, respectively (Fig. 7h). Multivariate Cox regression analyses confirmed that the risk score was an independent prognostic factor for GBM, surpassing other common clinical characteristics (Fig. 7i, j). Specifically, in the TCGA cohort, multivariate Cox analyses indicated that the risk score was an independent prognostic factor regardless of age, race, and gender (Fig. 7i). In the CGGA cohort, it was an independent prognostic factor irrespective of age and gender (Fig. 7j). To explore the clinical utility of the risk model, a nomogram incorporating age, gender, and risk score was developed to predict OS for GBM patients based on both the TCGA and CGGA cohorts (Fig. 7k–m). The analysis revealed that the risk score had the most substantial impact on predicting OS, suggesting that prognosis for GBM can be effectively predicted using the risk model based on *TNFRSF12A* and *SFN*. Calibration curves for 1 and 2 years demonstrated a reasonable alignment between expected and observed values (Fig. 7l–n). The analysis established and validated a risk-scoring model for GBM using six TSGs, with *TNFRSF12A* and *SFN* emerging as independent prognostic factors. This model accurately predicts OS in both TCGA and CGGA cohorts, demonstrating superior performance over traditional clinical factors and highlighting the risk score’s substantial impact on survival prediction, as supported by calibration curves aligning expected and observed values.

### TNFRSF12A in both AD and GBM

**Fig. 8:**
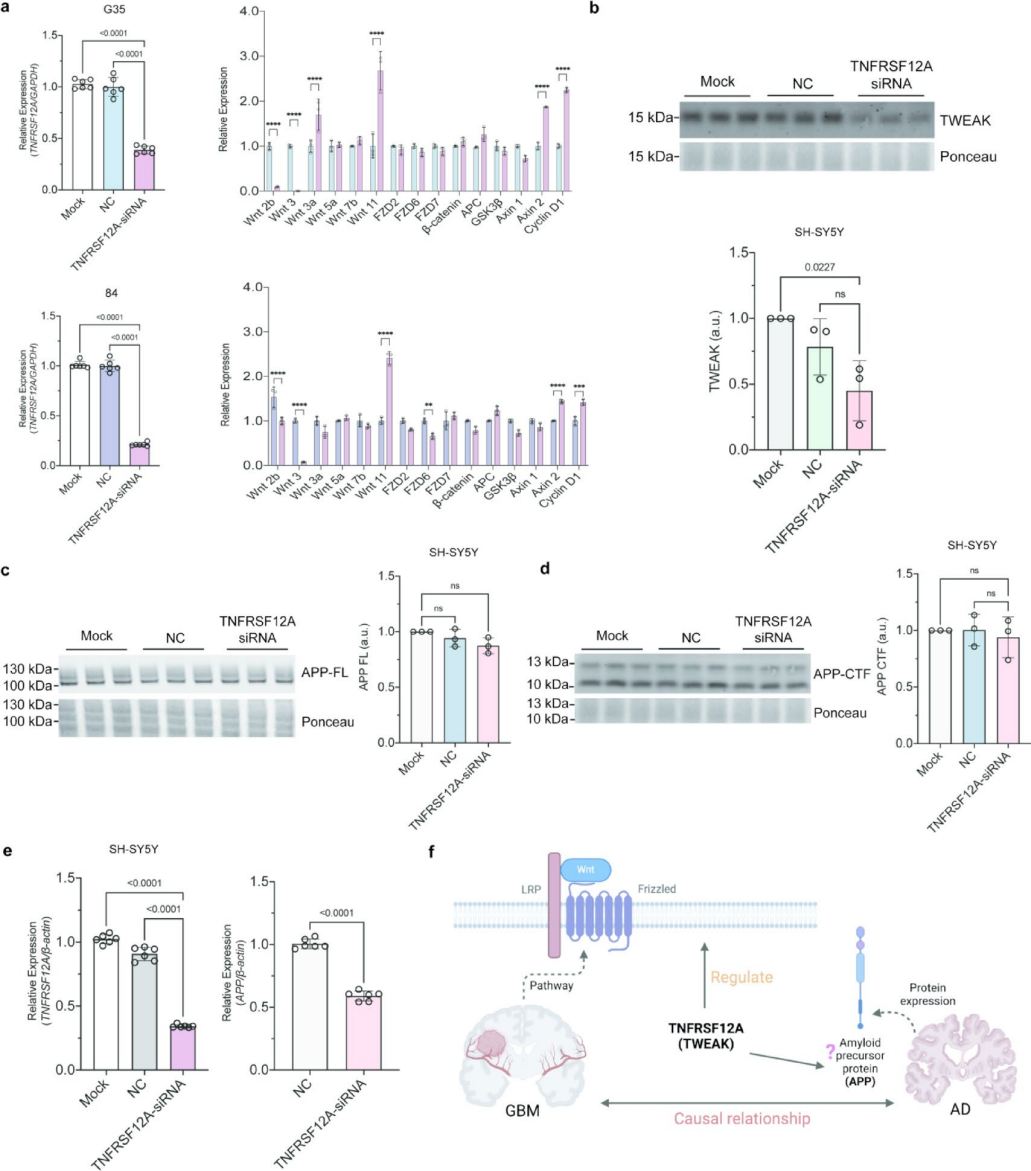
*TNFRSF12A* in both AD and GBM. (**a**) Relative mRNA expression levels of Wnt pathway genes in GBM cell lines (G35 and 84) following successful *TNFRSF12A* knockdown. Non-transfected GBM cells lines were used as the mock group (Mock), while cells transfected with negative siRNA served as the negative control (NC). Results represent data from three separate experiments. Data are presented as mean ± standard deviation (SD) (n = 3 biological replicates). Significance levels were determined using two-way ANOVA with Bonferroni’s post-hoc test (*P < 0.05; ** *P* < 0.01; *** *P* < 0.001; **** *P* < 0.0001). (**b**) Western blot analysis (top panel) and quantification (bottom panel) of TWEAK protein expression in SH-SY5Y cells with or without *TNFRSF12A*-siRNA. Band intensities were normalized to ponceau (total protein) for each sample and to the average of the control group (Mock). Non-transfected SH-SY5Y cells were used as the mock group (Mock), while cells transfected with negative siRNA served as the negative control (NC). Representative images are shown. Data are presented as mean ± SD (n = 3 biological replicates). Statistical significance was assessed using two-way ANOVA with Bonferroni’s post-hoc test (*P* < 0.05; ns, no significance). (**c**, **d**) Western blot analysis (left) and quantification (right) of full-length amyloid precursor protein (APP-FL) and its C-terminal fragment (APP-CTF) in SH-SY5Y cells with or without *TNFRSF12A*-siRNA. Band intensities were normalized to ponceau (total protein) and to the Mock group’s average. Non-transfected SH-SY5Y cells were used as Mock, while cells transfected with negative siRNA served as NC. Representative images are shown. Data are presented as mean ± SEM (n = 3). Statistical significance was determined using one-way ANOVA with Bonferroni’s post-hoc test (*P* < 0.05; ns, no significance). (**e**) Relative mRNA expression levels of *APP* gene in the SH-SY5Y cell line following successful *TNFRSF12A* knockdown. Data are presented as mean ± SD (n = 6). Statistical significance was determined using an unpaired Student’s t-test (*P* < 0.05). (**f**) Schematic diagram of the mechanism of *TNFRSF12A* in GBM and AD.

Through the analysis of TSGs in both AD and GBM, we identified *TNFRSF12A* as a significant factor in both conditions. For molecular validation, we used TNFRSF12A siRNA to knock down *TNFRSF12A* in GBM cell lines (G35 and 84) and SH-SY5Y cells over- expressing APP. We observed that siRNA- *TNFRSF12A* significantly downregulate the expression of *TNFRSF12A* RNA, however not as a complete loss of transcript in GBM (Fig. 8a) and SH-SY5Y cells (Fig. 8e). In GBM cells, we next investigated the impact of partial knockdown on the expression of genes involved in the key pathway in GBM (Wnt pathway). In G35, *Wnt 2b* and *Wnt 3* expression decreased (*P* < 0.0001), while *Wnt 3a*, *Wnt 11*, *Axin 2*, and *Cyclin D1* increased (*P* < 0.0001). In 84, *Wnt 2b*, *Wnt 3* (*P* < 0.0001), and *FZD6* (*P* < 0.01) decreased, while *Wnt 11*, *Axin 2*, and *Cyclin D1* increased (*P* < 0.0001) (Fig. 8a). We next investigated the effect of TNFRSF12A downregulation on the expression of APP. We first confirmed partial knockdown of TWEAK ( a type II transmembrane protein encoded by the *TNFRSF12A* gene, and a tumor necrosis factor-like weak inducer of apoptosis)^34^ in SH-SY5Y cells (*P* < 0.05) (Fig. 8b). However, levels of full-length amyloid precursor protein (APP-FL) and its C-terminal fragment (APP-CTF) were not significantly decreased by downregulation of TWEAK (Fig. 8c, d). qPCR revealed decreased *APP* mRNA levels upon *TNFRSF12A* knockdown (*P* < 0.0001) (Fig. 8e). In summary, *TNFRSF12A* can regulate the Wnt pathway in GBM and reduce the expression of *APP* at the transcription level (Fig. 8f).

## Discussion

The inverse relationship between cancer and NDDs has long intrigued researchers^35^. In this study, we explored this dichotomy by focusing on AD and GBM^36–38^—two disorders of the CNS that reflect opposing cellular fates: progressive neuronal loss versus uncontrolled proliferation. By integrating MR, bulk and single-cell transcriptomics, and experimental knockdown assays, we provide multi-level evidence supporting the role of TSGs in linking these two disease states.

Our bidirectional MR analysis revealed a statistically supported inverse genetic association between AD and GBM, consistent with previous population-based studies and recent MR reports identifying inverse associations between glioma and neurodegenerative traits^4^. The observation that individuals with a parental history of AD had reduced GBM risk reinforces the hypothesis that shared heritable factors may exert divergent effects depending on disease context. These results add genetic weight to the long-standing epidemiological hypothesis of antagonistic pleiotropy between cancer and NDDs.

At the cellular level, scRNA-seq analysis revealed that GBM tissues exhibited more complex and robust cell–cell communication networks than AD, particularly among astrocytes, neurons, and endothelial cells. This aligns with prior descriptions of tumor microenvironments as rich in pro-growth and pro-angiogenic signaling. Conversely, signaling in AD was more confined and neuron-centric, echoing reports of synaptic degeneration and glial activation as key disease features^39^. Differential pathway activation, including upregulation of Wnt signaling in GBM and APP-related processes in AD, reflects how shared molecular axes can diverge under distinct pathological pressures. Nevertheless, we fully acknowledge that these observations may be influenced by sample imbalance, which represents a limitation of the current study. Future investigations with larger and more balanced cohorts will be crucial to validate and generalize these findings across broader patient populations.

Focusing on TSGs, we identified 413 shared genes between AD and GBM, with a substantial proportion showing inverse expression patterns. Some of these, such as *PEG3* and *NUPR1*, have been individually implicated in neuronal survival and tumor suppression, respectively^40,41^. The systematic contrast of these TSGs across diseases supports the idea of transcriptional antagonism.

Moreover, we demonstrated that key TSGs like *CDCP1*, *PDLIM4*, and *TNFRSF12A* may serve as therapeutic targets for AD, while *TNFRSF12A* and *SFN* are promising for GBM. This dual role of *TNFRSF12A* in AD and GBM supports the hypothesis of an overlap relationship between cancer and NDDs, offering new avenues for targeted therapies. The *TNFRSF12A* gene, part of the tumor necrosis factor receptor superfamily, plays a key role in molecular pathways linked to various cancers. In gastric cancer, *TNFRSF12A* activates the PI3K/Akt and NF-kB signaling pathways, contributing to tumor development^42,43^. Similarly, it triggers the JAK/STAT pathway in non-small cell lung cancer^44^ and the NF-kB pathway in prostate cancer^45,46^, promoting tumor cell invasion and migration. Notably, *TNFRSF12A* is significantly overexpressed in gliomas, enhancing cell migration and invasion, thus reinforcing its tumor-promoting effects^47,48^. Here it is important to mention that we attempt to knock down *TNFRSF12A* using siRNA, however, did not obtained complete knockdown. Nevertheless, with partial knockdown we could show certain changes in genes associated with Wnt signaling pathway which are pre-requisite for GBM cells proliferation, and a significant downregulation of *APP* mRNA levels in SHSY5Y cells. Levels of APP (protein) tended to be decreased in cells upon partial downregulation of TWEAK, but the differences were not statistically significant. These findings suggest a potential regulatory role for *TNFRSF12A* in modulating both Wnt and APP signalling pathways. However, further studies—such as CRISPR/Cas9-mediated gene knockout or rescue experiments—are required to establish a causal relationship.

It is important to distinguish between established findings and hypothesis-generating observations. While MR and transcriptomic results offer robust statistical evidence, the role of *TNFRSF12A* in APP metabolism remains speculative and requires further mechanistic validation. Moreover, several limitations should be acknowledged. First, functional assays were limited to in vitro partial knockdown models. Second, transcriptomic data were cross-sectional and limited to publicly available cohorts from predominantly European populations. Third, our analysis centered on *TNFRSF12A*, yet other candidate genes such as *CDCP1*, *SFN*, and *PDLIM4* also demonstrated disease-specific expression and may warrant future investigation.

Clinically, *TNFRSF12A* holds promise as a dual-disease biomarker and potential therapeutic target. Its consistent dysregulation in both AD and GBM suggests a shared pathogenic axis that could be leveraged for diagnostic and therapeutic innovation. Notably, *TNFRSF12A* (also known as Fn14) is a membrane-bound receptor with emerging druggability, as several preclinical studies have explored monoclonal antibodies and antibody–drug conjugates targeting this pathway, particularly in oncology^49–51^. From a diagnostic perspective, its expression can be detected using established platforms such as immunohistochemistry, in situ hybridization, and transcriptomic profiling. As gene-targeted therapies and ligand-modulating strategies advance, the clinical translation of *TNFRSF12A* may enable precision interventions that address inflammatory, proliferative, and degenerative states across central nervous system disorders.

This study provides molecular and transcriptomic evidence supporting an inverse relationship between AD and GBM. Through integrative analyses of genetic causality, cell-specific gene expression, and intercellular communication, we identified TSGs that display opposing activity patterns in neurodegeneration and tumorigenesis. Among them, *TNFRSF12A* emerged as a candidate of dual relevance, functionally linked to both Wnt-driven proliferation in GBM and APP regulation in AD. Our findings not only underscore the importance of TSGs as disease modifiers across the CNS spectrum, but also introduce *TNFRSF12A* as a potential cross-disease biomarker or therapeutic target. These insights lay the foundation for future mechanistic studies, and suggest the possibility of shared molecular targets for two seemingly unrelated brain pathologies. By highlighting the dual roles of TSGs like *TNFRSF12A*, this study opens new avenues for precision medicine that transcend traditional boundaries between oncology and neurology.

## Material and methods

### Mendelian Randomization analysis

To investigate potential causal relationships among AD, GBM, and parental history of AD, we conducted bidirectional two-sample MR using the “TwoSampleMR” R package (v0.5.6). MR infers causality from genetic associations by using single nucleotide polymorphisms (SNPs) as instrumental variables.GWAS summary statistics were retrieved from the IEU OpenGWAS Project (https://gwas.mrcieu.ac.uk/) (Table 1) ^52^:

AD: ebi-a-GCST002245, 17,008 cases and 37,154 controls, 7,022,150 SNPs^53^.

GBM: finn-b-C3_GBM_EXALLC, 91 cases and 174,006 controls, 16,380,303 SNPs (https://gwas.mrcieu.ac.uk/datasets/finn-b-C3_GBM/).

Parental AD history: ebi-a-GCST90013921, 407,089 individuals, 11,039,079 SNPs^54^.

All participants were of European ancestry. Five MR methods were applied: inverse variance weighting (IVW), weighted median, MR-Egger regression, maximum likelihood, and radial IVW. Instrument validity was assessed via Cochran’s Q test for heterogeneity^55^ and MR-Egger intercept^56^ for pleiotropy (*P* < 0.05 indicating potential bias). Causal relationships were considered significant if at least one method yielded *P* < 0.05.

### Source of raw data

The AD and GBM single-cell data sets GSE175814 and GSE223065 were downloaded from the NCBI Gene Expression Omnibus database (https://www.ncbi.nlm. nih.gov/geo/), including 2 AD samples and 6 GBM tumor samples. These datasets were selected because they contain high-quality 10 × Genomics data with well-annotated brain cell types and sufficient cell numbers to allow meaningful clustering and downstream analysis. The bulk RNA-seq data of patients with AD, GSE122063, GSE37263 and GSE63061, were downloaded from the NCBI GEO database. GSE122063 served as the discovery dataset due to its well-matched AD and control samples from brain cortex tissue. GSE37263 and GSE63061 were chosen as external validation datasets based on their large sample sizes and use of independent microarray platforms, enabling cross-platform validation. The GSE122063 dataset was generated using the GPL16699 Agilent-039494 SurePrint G3 Human GE v2 8 × 60 K Microarray. The GSE37263 dataset was generated using GPL5175 Affymetrix Human Exon 1.0 ST Array. The GSE63061 dataset was generated using GPL10558 Illumina HumanHT-12 V4.0 expression beadchip. GSE122063 dataset consisted of 12 brain cortex samples from AD patients and 11 normal brain cortex samples from controls. GSE37263 and GSE63061 datasets, which served as external validation cohorts, consisted of 147 AD samples and 142 control samples. The RNAseq data, mutation data, and clinicopathological information for TCGA-GBM, which includes 145 glioblastoma samples, were obtained from the UCSC Xena website (https://xena.ucsc.edu/). TCGA was chosen for its comprehensive molecular and clinical characterization, representing the gold standard in GBM research. A validation model was created using gene expression data obtained from the China Glioma Genome Atlas (CGGA) data portal (http://www.cgga.org.cn) with 656 glioma patients. The expression data was obtained in TPM format. The process of batch correction and integration of the two sets of gene expression data was performed using the “limma” package in R. Differential gene expression analysis was performed on both AD and GBM cohorts using the “limma” package in R, with | log2FC |> 1.0 and FDR (false discovery rate) < 0.05 as the thresholds.

### Single-cell data preprocessing

Quality control was conducted in “Seurat” package (version 4.1.1). Cells expressing < 300 or > 9,000 genes, or with mitochondrial gene content > 30%, were excluded. Genes expressed in < 3 cells were also filtered. The expression matrix underwent normalization using the “NormalizeData” function. The batch impact was decreased using the “RunHarmony” function from the “harmony” package^57^. The process of dimension reduction begins by using the FindVariableFeatures tool to identify and exclude 2000 genes that exhibit high variability. Subsequently, Principal Component Analysis (PCA) was conducted on the set of genes that exhibited significant variability. We created a shared closest neighbor network using the FindTNeighbor function and then detected clusters using the FindClusters function. Subsequently, the outcomes were shown using the uniform manifold approximation and projection (UMAP) techniques. Afterwards, clusters were labeled using classic cell surface markers.

### Cell–cell communication analysis of AD and GBM

To analyze cell–cell communication in AD and GBM, we utilized the R package “CellChat” version 1.6.1^58^, which integrates scRNA-seq data with the CellChatDB ligand-receptor interaction database. We applied CellChat separately to AD and GBM datasets (GSE175814 and GSE223065) to infer communication networks across key cell types, including astrocytes, neurons, microglia, oligodendrocytes, and endothelial cells. To compare these networks, we merged the CellChat objects from both conditions, allowing us to identify significant changes in interactions between cell populations. We visualized differential interactions using bar plots and heatmaps generated with the “ComplexHeatmap” R package version 2.16 ^59^. To pinpoint altered signaling sources and targets, we conducted network centrality analysis by calculating the outdegree (signaling sources) and indegree (signaling targets) centralities for each cell population. We then visualized the differential centralities in a 2D space, highlighting cell populations with significant changes in their signaling activity between the two conditions. Positive and negative values indicate increased or decreased signaling in GBM compared to AD, respectively.

### Differentially expressed TSGs function enrichmen

1217 TSGs were obtained from TSGs database (https://bioinfo.uth.edu/TSGene/). The screening of Differentially expressed TSG among cell types was conducted using the “FindAllMarkers” function from the “Seurat” package (*P* < 0.05 and | log2FC |> 0.25). The biological functions of differentially expressed TSG were explored by Gene Ontology (GO) and visualized with “ClusterGVis” package.

### TSG-based grouping and AUCell scoring

The TSG score was calculated by “AUCell” R package (version 1.16.0) for each cell in scRNA-seq data. The high- and low-score cells as well as patients were grouped, respectively, by the median of the corresponding scores. Differentially expressed genes (DEGs) between the high- and low-score groups were identified using the “FindAllMarkers” function at the cell level.

### Gene set enrichment analysis (GSEA)

The “clusterProfiler” package of R software was used to carry out the functional enrichment analysis, which included KEGG and GO analysis. We adjusted the *P* values using the Benjamini—Hochberg (BH). The GSEA was utilized to identify genes that had statistically significant and consistent alterations between two biological states. Genes with a corrected *P* value and false discovery rate (FDR) below 0.05 were considered to be significant.

### Characteristic genes in AD

We applied LASSO regression and Random Forest to identify key AD-related genes, as both are widely used feature selection methods that complement each other: LASSO performs variable shrinkage and selects features by penalizing the absolute size of the regression coefficients, while Random Forest captures non-linear relationships and evaluates feature importance based on ensemble decision trees^60^. LASSO analysis was conducted using the glmnet package with tenfold cross-validation to determine the optimal value of the regularization parameter (λ) that minimized the mean cross-validated error. Genes with non-zero coefficients at the optimal λ were retained as significant features. For Random Forest (RF), we used the “randomForest” package to construct 500 trees and ranked features based on the Mean Decrease in Gini index. To ensure robustness, tenfold cross-validation was performed, and the top genes consistently showing high importance scores across folds were selected. Ultimately, the intersection of results from both methods identified 3 genes with the highest predictive relevance, which were used for further analysis.

### Development and validation of prognostic features in GBM

The putative hub TSG with prognostic significance were mostly discovered by univariate Cox analysis. Afterwards, the prognostic hub selected TSG underwent LASSO regression analysis, with the “glmnet” package in R being used to minimize the number of genes in the final risk model. Ultimately, a TSG signature consisting of 2 genes was identified. The regression coefficient for these genes were determined using stepwise multivariate Cox analysis. A TSG risk score system for samples was devised as follows:

$$Risk Score = \beta_{1} \times ExpressionSEN + \beta_{2} \times ExpressionTNFRSF12A$$

All patients were divided into high- and low-risk groups based on their median risk score. The R packages “survminer” and “ggrisk” were utilized to generate survival curves and risk maps, illustrating differences in survival rates and patient statuses. Additionally, an additional external cohort known as the CGGA cohort was utilized to assess the efficacy of the prognostic model. A nomogram was constructed utilizing a risk score and clinicopathological characteristics. The net clinical benefit of the models was evaluated using Decision Curve Analysis (DCA) with the “ggDCA” package in R^61^.

### Cell culture and transfections

Two primary GBM cell lines (G35, 84), provided by Professor Jarek Maciaczyk, were cultured at 37 °C in 5% CO₂. Both lines were IDH wildtype and maintained in DMEM (Gibco, USA, Cat. No: 11965092) with FBS (Gibco, USA, Cat. No: 110270106) and penicillin/streptomycin (Gibco, USA, Cat. No: 15140122). Cells were passaged at 80–90% confluency using 0.5% trypsin–EDTA (Gibco, USA, Cat. No: 15400054). SH-SY5Y human neuroblastoma cells (ATCC, USA, CTR-2266), stably transfected with wild-type APP695 (Swedish mutation), were cultured in DMEM/F-12 (Life Technologies, USA, Cat. No: 10565018) with 10% FBS, 1% PS, 2 μg/ml G418 (Gibco, USA, Cat. No: 10131035), 2 mM L-glutamine, 1% sodium pyruvate (Life Technologies, USA, Cat. No: 11360039), and 1% non-essential amino acids (Life Technologies, USA, Cat. No: 11140050). SH-SY5Y cells expressing APP were maintained at 37 °C in 5% CO₂.

For gene silencing, cells were transfected with siRNA targeting *TNFRSF12A* (IDT, Germany, Cat. No: CD.Ri.478496.13.1) using Lipofectamine™ RNAiMAX (Invitrogen, USA, Cat. No: 13778–150) according to the manufacturer’s protocol. Briefly, cells were seeded in 6-well plates at a density of 5 × 10^5^ cells per well and allowed to adhere overnight. The next day, the mixture of Lipofectamine™ RNAiMAX with Opti-MEM® (Gibco, USA, Cat. No: 11524456) was firstly incubated for 5 min at RT, then mixed with 10 μM siRNA (diluted in Opti-MEM) and together incubated for 20 min at RT. The transfection mixture was then added dropwise to the cells. Cells were harvested at 48 h for downstream analyses.

### RT-qPCR

RNA extraction and quantitative polymerase chain reaction. RNA isolation of GBM G35, GBM 84 and SH-SY5Y samples was performed with RNeasy Plus Mini Kit (QIAGEN, Germany, Cat. No: 74136). Complementary DNA was synthesized by reverse transcription using High-Capacity cDNA Reverse Transcription Kit (Thermo Fisher Scientific, USA, Cat. No: 4368814). Quantitative polymerase chain reaction was performed on genes related to Wnt/beta-catenin signaling pathway (*Wnt 2b*, *Wnt 3*, *Wnt 3a*, *Wnt 5a*, *Wnt 7b*, *Wnt 11*, *FZD2*, *FZD6*, *FZD7*, *β-catenin*, *APC*, *GSK3β*, *Axin 1*, *Axin 2* and *Cyclin D1*), *TNFRSF12A* and *APP* (Qiagen, Germany, Cat. No: QT01886815) using PowerTrack™ SYBR Green Mastermix (Thermo Fisher Scientific, USA, Cat. No: A46109). *GAPDH* and *β-actin* was selected as the internal reference gene. The primer sequence is as follows (Supplementary Table 1) . The Δ–Δ Ct (2–∆∆Ct) approach was used to measure the relative expression levels of target genes, which were standardized against *GAPDH* or *β-actin* mRNA levels.

### Preparation of cellular membrane fractions

For preparation of cellular membranes, as described before^62^, cells were washed once with ice cold PBS, scraped in an appropriate volume of hypotonic buffer (10 mM Tris–HCl, 1 mM EDTA, 1 mM EGTA in dH2O, pH 7.6) supplemented with protease inhibitor cocktail (Sigma-Aldrich, USA, Cat. No: 04693116001) and collected in tubes. Cells were incubated on ice for 10 min and subsequently homogenized using a syringe with a 0.6 mm cannula, drawing 20 times. After centrifugation for 10 min at 200×*g* at 4 °C, the resultant supernatant was transferred into a fresh tube and centrifuged for 1 h at 16,000×*g* and 4 °C. The supernatant was removed and the remaining pellet considered as a crude membrane fraction. For extraction of proteins, the membrane fraction was lysed in STEN lysis buffer (150 mM NaCl, 50 mM Tris, 2 mM EDTA, 1% NP-40, 1% Triton X-100 in dH2O, pH 7.4) supplemented with protease inhibitor cocktail (Sigma-Aldrich, USA, Cat. No: 4906837001), and incubated for 20 min on ice. The lysate was centrifuged for 10 min at 16,000×*g* at 4 °C and the supernatant used for further direct analysis by SDS-PAGE and western immunoblotting.

### Sodium dodecyl-sulfate polyacrylamide gel electrophoresis (SDS-PAGE) and western immunoblotting

Samples were mixed with SDS sample buffer and heated for 5 min at 95 °C. Proteins were separated using pre-cast NuPAGE Novex Bis–Tris Gels 4–12% (Invitrogen, USA), NuPAGE running chambers, and NuPAGE MES SDS Running Buffer (Invitrogen, USA, Cat. No: NP0002) at 150 V. Proteins were transferred onto nitrocellulose membranes by wet transfer technique at a constant current of 400 mA for 1 h and 45 min. After transfer, stain the membrane with Ponceau S solution for 5 min at room temperature. Rinse the membrane with distilled water until clear bands are visible. Document the stained membrane, then proceed with destaining using Tris-Buffered Saline, 0.1% Tween® 20 Detergent (TBST) for Western blotting. After blocking for 1 h with constant agitation in TBST containing 5% milk powder, membranes were incubated over night at 4 °C with primary antibodies diluted in TBST. Membranes were washed 3 times for 5 min, and incubated in TBST containing the respective secondary antibody conjugated a fluorophore (IRDye800CW, 680RD, Li-COR Biosciences, Germany) for 1 h at RT, detailed information on the antibodies used is available in Supplementary Table 2. After washing the membrane 3 times for 5 min, signals were detected using an Odyssey® CLx (Li-COR Biosciences, Germany). ImageJ (NIH, USA) was used for quantitative analysis of the blots.

### Statistical analysis

Unless otherwise specified, analyses were conducted using the statistical package in R version 4.3.1. Univariate and multivariate Cox regression (R package “survival”) analyses were used to identify clinical traits with prognostic potential in the high- and low-risk groups. Pearson correlation analysis was used to assess correlation coefficients. Wilcoxon signed-rank test was carried out for statistical comparisons between two groups. Statistical analysis was performed using GraphPad Prism (version 8.0). Experimental data are presented as means ± SD. An unpaired Student’s t-test or two-way analysis of variance (ANOVA) with Bonferroni’s post-hoc test was performed to analyze statistical significance. *P* < 0.05 was considered statistically significant.

## Electronic supplementary material

Below is the link to the electronic supplementary material.

Supplementary Material 1

Supplementary Material 2

Supplementary Material 3

## Data Availability

All data needed to evaluate the conclusions in the paperware present in the paper and/or the Supporting Information. The data sets generated during and/or analyzed during the current study are available from the corresponding author on request.
