# Supplementary material for: Potential role of TNFRSF12A in linking glioblastoma and alzheimer’s disease via shared tumour suppressor pathways
Source: Sci Rep. 2025 Jul 1;15:21535. doi: 10.1038/s41598-025-08000-7 (PMC12215723; doi:10.1038/s41598-025-08000-7)
Supplement: Supplementary file 1 — Supplementary Material 1 [file 41598_2025_8000_MOESM1_ESM.docx]

**Supplementary** **table 1. Detailed information on the primer sequence**

| **Primer name** | **Forward sequence** | **Reverse sequence** |
| --- | --- | --- |
| Wnt2b | 5′-GCCGTGTCATGCTCAGAA-3′ | 5′-GTGGACTACCCCTGCTGATG-3′ |
| Wnt3 | 5′-CTCGCTGGCTACCCAATTT-3′ | 5′-GCCCAGAGATGTGTACTGCTG-3′ |
| Wnt3a | 5′-CATGAACCGCCACAACAAC-3′ | 5′-TGGCACTTGCACTTGAGGT-3′ |
| Wnt5a | 5′-ATTGTACTGCAGGTGTACCTTAAAAC-3′ | 5′-CCCCCTTATAAATGCAACTGTTC-3′ |
| Wnt7b | 5′-CGCCTCATGAACCTGCATA-3′ | 5′-GCTGCATCCGGTCCTCTA-3′ |
| Wnt11 | 5′-TGTGCTATGGCATCAAGTGG-3′ | 5′-CAGTGTTGCGTCTGGTTCAG-3′ |
| FZD2 | 5′-GGTGTCGGTGGCCTACAT-3′ | 5′-GAGAAGCGCTCGTTGCAC-3′ |
| FZD6 | 5′-TGGGTTGGAAGCAAAAAGAC-3′ | 5′-TCTTCGACTTTCACTGATTGGA-3′ |
| FZD7 | 5′-GCCAGCTTGTGCCTAATAGAA-3′ | 5′-AGCCGGGAGAAACTCACAG-3′ |
| β-catenin | 5′-CTTACACCCACCATCCCACT-3′ | 5′-CCTCCACAAATTGCTGCTGT-3′ |
| APC | 5′-GCCCCTGACCAAAAAGGAAC-3′ | 5′-TGGCAGCAACAGTCCCACTA-3′ |
| GSK3β | 5′-CAAGCCAAACTTTGTGACTCAG-3′ | 5′-TATCAGGATCCAGCAAGAGGTT-3′ |
| Axin1 | 5′-AGCCGTGTCGGACATGGA-3′ | 5′-AAGTAGTACGCCACAACGATGCT-3′ |
| Axin2 | 5′-TGTGAGGTCCACGGAAACTG-3′ | 5′-CGTCAGCGCATCACTGGATA-3′ |
| Cyclin D1 | 5′-TCAAATGTGTGCAGAAGGAGGT-3′ | 5′-GACAGGAAGCGGTCCAGGTA-3′ |
| TNFRSF12A | 5′-CTCTGAGCCTGACCTTCGTG-3′ | 5′-GTCTCCTCTATGGGGGTGGT-3′ |
| GAPDH | 5′-TTCCAGCCTTCCTTCCTGGG-3′ | 5′-TTGCGCTCAGGAGGAGCAAT-3′ |
| β-actin | 5′-CCAACCGCGAGAAGATGA-3′ | 5′-CCAGAGGCGTACAGGGATAG-3′ |

**Supplementary table 2. Detailed information on the antibodies**

| **Antibodies** | **Reference or source** | **Identifier or catalog number** |
| --- | --- | --- |
| **Primary antibodies** | | |
| Anti-Amyloid-Precursor- Protein (APP) (human, mouse and rat) | Biolegend | Cat# BLD- 802,801 Clone: C1/6.1 |
| TWEAK Receptor/Fn14 Antibody (human, mouse and rat) | Cell Signaling Technology | Cat# 4403S |
| **Secondary antibodies** | | |
| IRDye680 RD donkey anti rabbit | Li-COR | Cat# 926-68073 |
| IRDye800CW donkey anti mouse | Li-COR | Cat# 926-32212 |
| IRDye800CW donkey anti rabbit | Li-COR | Cat# 926-32213 |
