# Supplementary material for: Potential role of TNFRSF12A in linking glioblastoma and alzheimer’s disease via shared tumour suppressor pathways
Source: Sci Rep. 2025 Jul 1;15:21535. doi: 10.1038/s41598-025-08000-7 (PMC12215723; doi:10.1038/s41598-025-08000-7)
Supplement: Supplementary file 2 — Supplementary Material 2 [file 41598_2025_8000_MOESM2_ESM.docx]

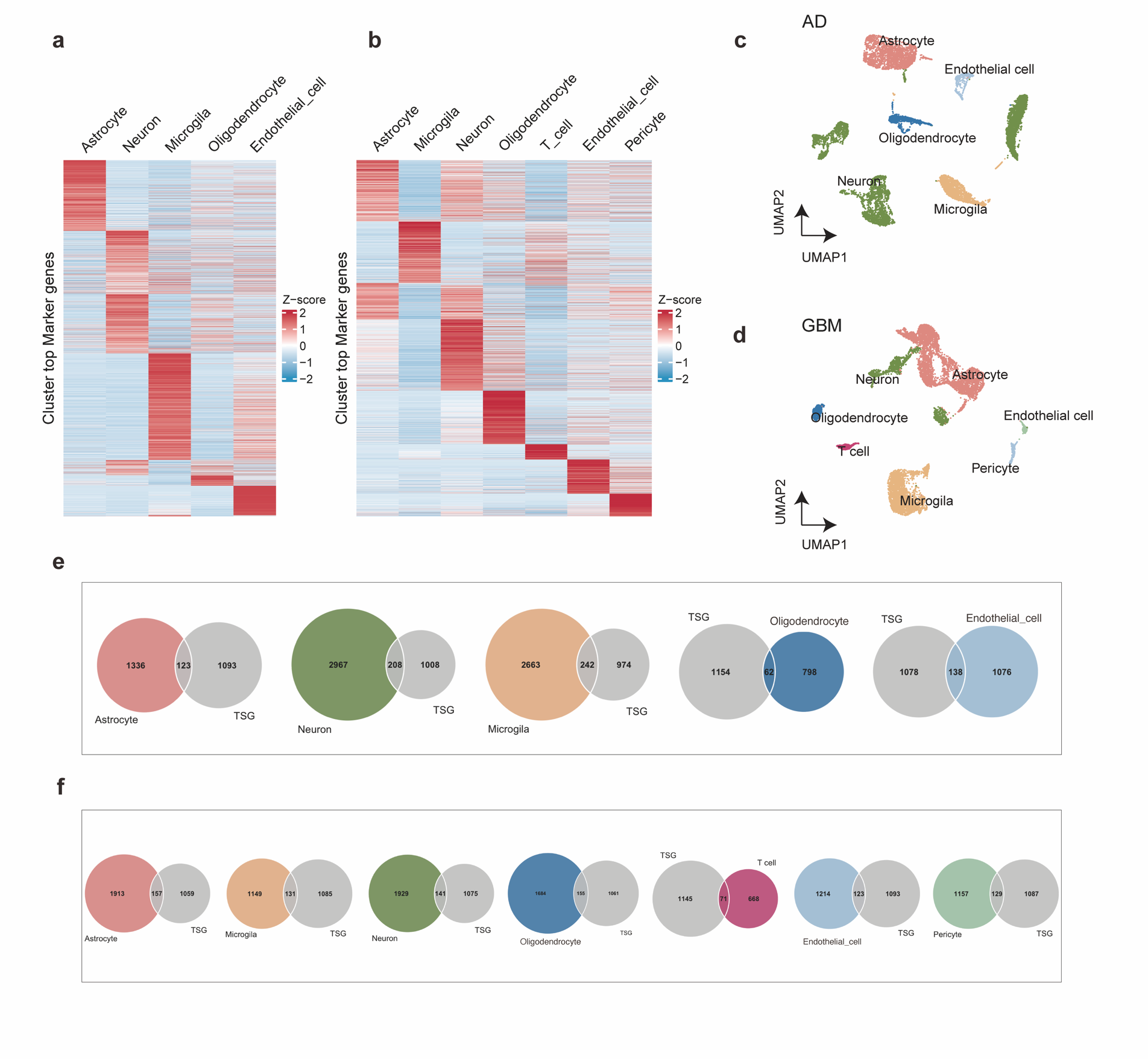


**Supplementary Fig. 1: Identification of major cell populations in AD and GBM.** The heatmap depicts the expression of marker genes in the cell types listed. **c-d** Uniform Manifold Approximation and Projection (UMAP) plot showing the annotation and color codes for 5 and 7 cell types respectively in the AD and GBM. **e** Intersection analysis of TSGs with specific cell types in AD. **f** Intersection analysis of TSGs with specific cell types in GBM.


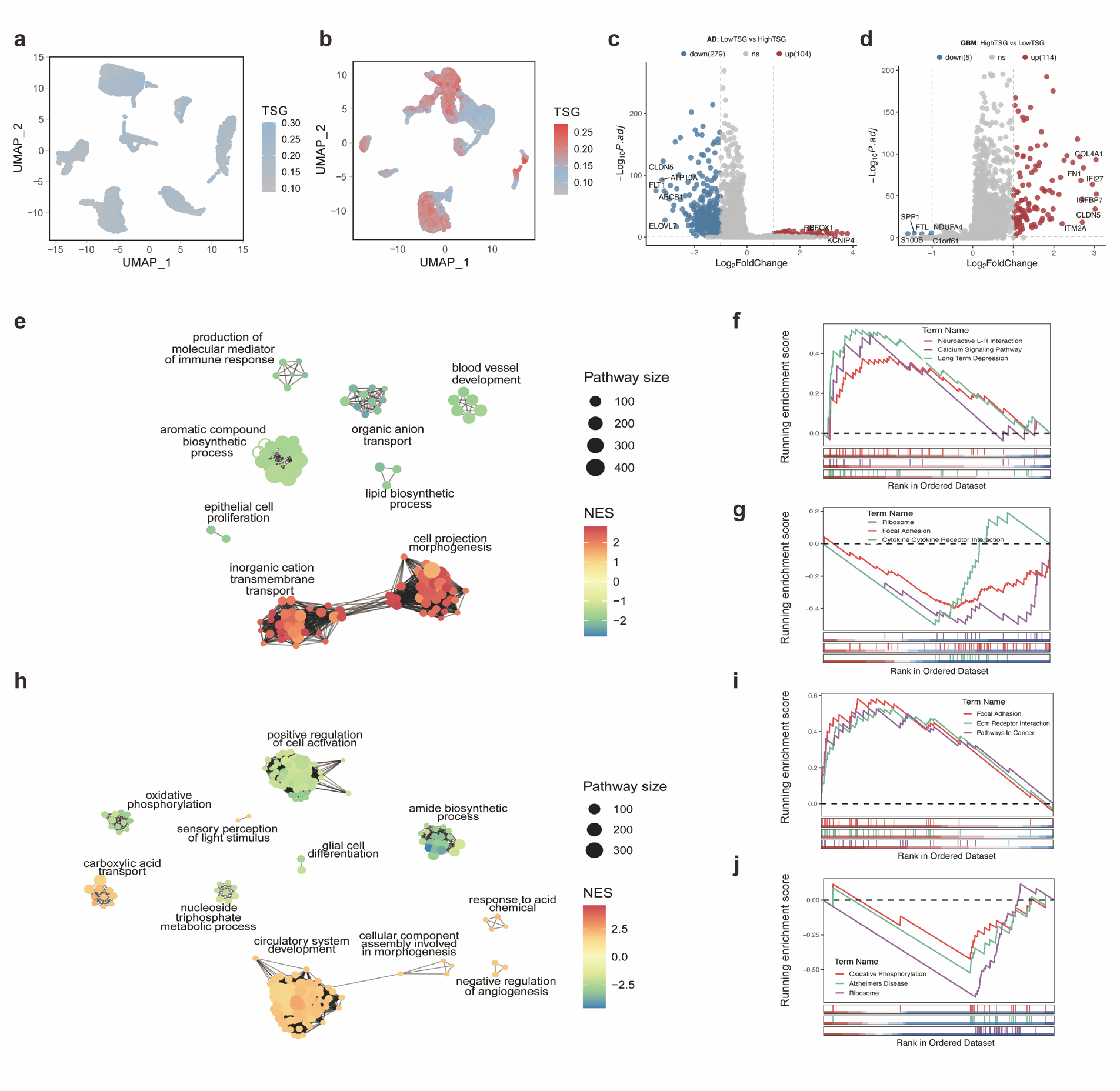


**Supplementary Fig. 2: Grouping of cells by tumor suppresser genes. a-b** Grouping of cells by shared TSGs in AD (**a**) and GBM (**b**). Each point corresponds to a single cell, positioned based on the similarity of its TSG expression profile to others. The color intensity of the points reflects the level of TSG expression. **c-d** Differential expression analysis between samples with low TSG expression versus those with high TSG expression in AD (**c**) and with high TSG expression versus those with low TSG expression in GBM (**d**) (P < 0.05). **e** Gene Ontology Biological Process (GOBP) function enrichment based on Gene Set Enrichment Analysis (GSEA) algorithm in the low TSG expression AD group. **f** Top 3 up-regulated KEGG enriched pathways based on GSEA in the low TSG expression AD group. **g** Top 3 down-regulated KEGG enriched pathways based on GSEA in the low TSG expression AD group. **h** GOBP function enrichment based on GSEA in the high TSG expression GBM group. **i** Top 3 up-regulated KEGG enriched pathways based on GSEA in the high TSG expression GBM group. **j** Top 3 down-regulated KEGG enriched pathways based on GSEA in the high TSG expression GBM group.

**Supplementary** **table 1. Detailed information on the primer sequence**

| **Primer name** | **Forward sequence** | **Reverse sequence** |
| --- | --- | --- |
| *Wnt2b* | 5′-GCCGTGTCATGCTCAGAA-3′ | 5′-GTGGACTACCCCTGCTGATG-3′ |
| *Wnt3* | 5′-CTCGCTGGCTACCCAATTT-3′ | 5′-GCCCAGAGATGTGTACTGCTG-3′ |
| *Wnt3a* | 5′-CATGAACCGCCACAACAAC-3′ | 5′-TGGCACTTGCACTTGAGGT-3′ |
| *Wnt5a* | 5′-ATTGTACTGCAGGTGTACCTTAAAAC-3′ | 5′-CCCCCTTATAAATGCAACTGTTC-3′ |
| *Wnt7b* | 5′-CGCCTCATGAACCTGCATA-3′ | 5′-GCTGCATCCGGTCCTCTA-3′ |
| *Wnt11* | 5′-TGTGCTATGGCATCAAGTGG-3′ | 5′-CAGTGTTGCGTCTGGTTCAG-3′ |
| *FZD2* | 5′-GGTGTCGGTGGCCTACAT-3′ | 5′-GAGAAGCGCTCGTTGCAC-3′ |
| *FZD6* | 5′-TGGGTTGGAAGCAAAAAGAC-3′ | 5′-TCTTCGACTTTCACTGATTGGA-3′ |
| *FZD7* | 5′-GCCAGCTTGTGCCTAATAGAA-3′ | 5′-AGCCGGGAGAAACTCACAG-3′ |
| *β-catenin* | 5′-CTTACACCCACCATCCCACT-3′ | 5′-CCTCCACAAATTGCTGCTGT-3′ |
| *APC* | 5′-GCCCCTGACCAAAAAGGAAC-3′ | 5′-TGGCAGCAACAGTCCCACTA-3′ |
| *GSK3β* | 5′-CAAGCCAAACTTTGTGACTCAG-3′ | 5′-TATCAGGATCCAGCAAGAGGTT-3′ |
| *Axin1* | 5′-AGCCGTGTCGGACATGGA-3′ | 5′-AAGTAGTACGCCACAACGATGCT-3′ |
| *Axin2* | 5′-TGTGAGGTCCACGGAAACTG-3′ | 5′-CGTCAGCGCATCACTGGATA-3′ |
| *Cyclin D1* | 5′-TCAAATGTGTGCAGAAGGAGGT-3′ | 5′-GACAGGAAGCGGTCCAGGTA-3′ |
| *TNFRSF12A* | 5′-CTCTGAGCCTGACCTTCGTG-3′ | 5′-GTCTCCTCTATGGGGGTGGT-3′ |
| *GAPDH* | 5′-TTCCAGCCTTCCTTCCTGGG-3′ | 5′-TTGCGCTCAGGAGGAGCAAT-3′ |
| *β-actin* | 5′-CCAACCGCGAGAAGATGA-3′ | 5′-CCAGAGGCGTACAGGGATAG-3′ |

**Supplementary table 2. Detailed information on the antibodies**

| **Antibodies** | **Reference or source** | **Identifier or catalog number** |
| --- | --- | --- |
| **Primary antibodies** | | |
| Anti-Amyloid-Precursor- Protein (APP) (human, mouse and rat) | Biolegend | Cat# BLD- 802,801 Clone: C1/6.1 |
| TWEAK Receptor/Fn14 Antibody (human, mouse and rat) | Cell Signaling Technology | Cat# 4403S |
| **Secondary antibodies** | | |
| IRDye680 RD donkey anti rabbit | Li-COR | Cat# 926-68073 |
| IRDye800CW donkey anti mouse | Li-COR | Cat# 926-32212 |
| IRDye800CW donkey anti rabbit | Li-COR | Cat# 926-32213 |
