# Supplementary figures and images for: Potential role of TNFRSF12A in linking glioblastoma and alzheimer’s disease via shared tumour suppressor pathways
Source: Sci Rep. 2025 Jul 1;15:21535. doi: 10.1038/s41598-025-08000-7 (PMC12215723; doi:10.1038/s41598-025-08000-7)

Figure 8b.

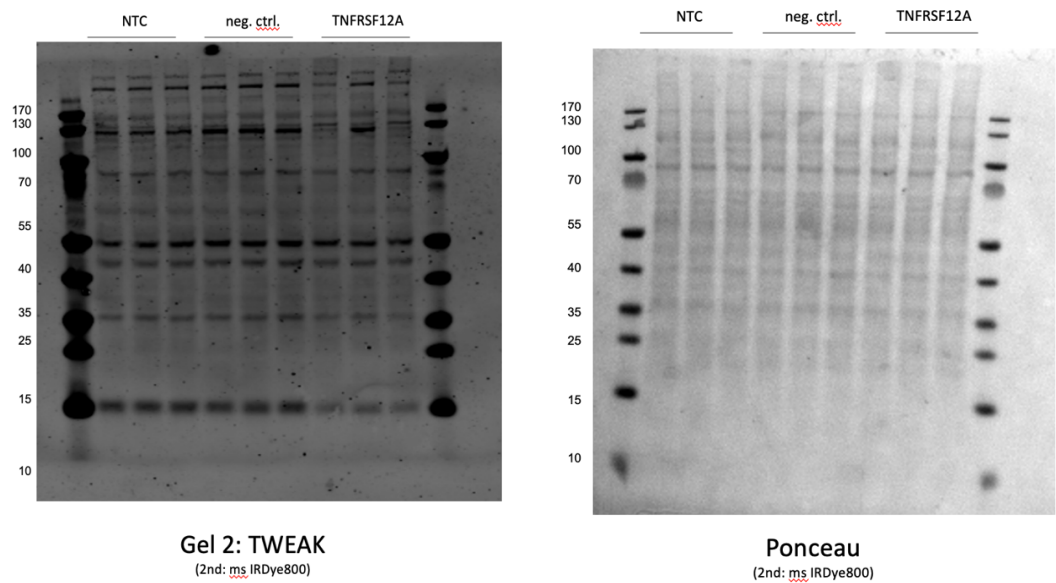

Figure 8c/8d.

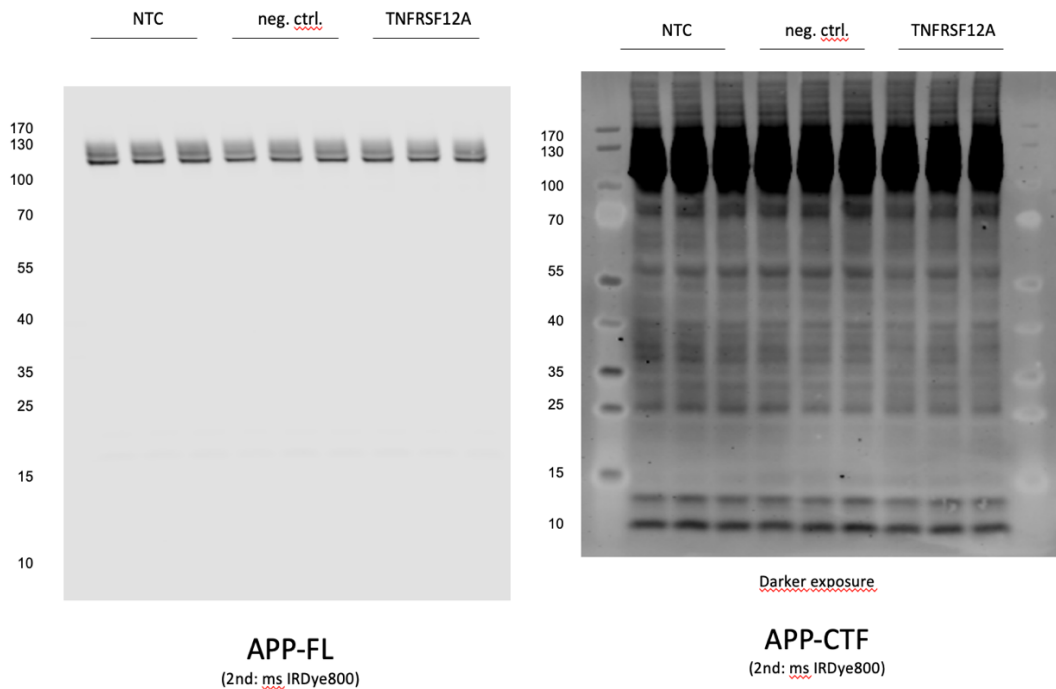

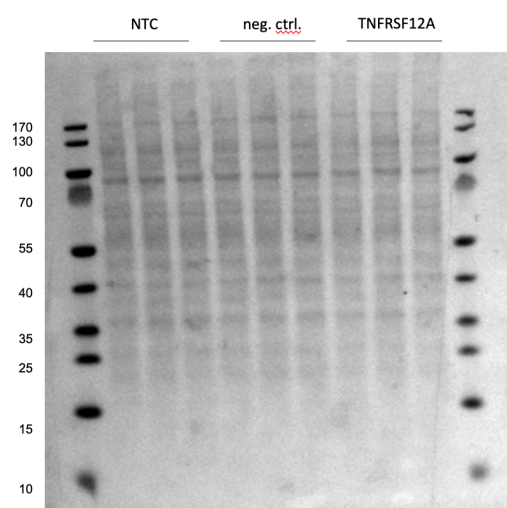

**Ponceau**  
(2nd: ms IRDye800)

Supplement: Supplementary file 3 — Supplementary Material 3 [file 41598_2025_8000_MOESM3_ESM.pdf]
